# Marked QTc Prolongation and Torsades de pointes in Patients with Chronic Inflammatory Arthritis

**DOI:** 10.3389/fcvm.2016.00031

**Published:** 2016-09-21

**Authors:** Pietro Enea Lazzerini, Pier Leopoldo Capecchi, Iacopo Bertolozzi, Gabriella Morozzi, Sauro Lorenzini, Antonella Simpatico, Enrico Selvi, Maria Romana Bacarelli, Maurizio Acampa, Deana Lazaro, Nabil El-Sherif, Mohamed Boutjdir, Franco Laghi-Pasini

**Affiliations:** ^1^Department of Medical Sciences, Surgery and Neurosciences, University of Siena, Siena, Italy; ^2^Cardiology Intensive Therapy Unit, Department of Internal Medicine, Hospital of Carrara, Carrara, Italy; ^3^VA New York Harbor Healthcare System, SUNY Downstate Medical Center, New York, NY, USA; ^4^NYU School of Medicine, New York, NY, USA

**Keywords:** torsades de pointes, chronic inflammatory arthritis, systemic inflammation, interleukin-6, rheumatoid arthritis, psoriatic arthritis, sudden death

## Abstract

Mounting evidence indicates that in chronic inflammatory arthritis (CIA), QTc prolongation is frequent and correlates with systemic inflammatory activation. Notably, basic studies demonstrated that inflammatory cytokines induce profound changes in potassium and calcium channels resulting in a prolonging effect on cardiomyocyte action potential duration, thus on the QT interval on the electrocardiogram. Moreover, it has been demonstrated that in rheumatoid arthritis (RA) patients, the risk of sudden cardiac death is significantly increased when compared to non-RA subjects. Conversely, to date no data are available about torsades de pointes (TdP) prevalence in CIA, and the few cases reported considered CIA only an incidental concomitant disease, not contributing factor to TdP development. We report three patients with active CIA developing marked QTc prolongation, in two cases complicated with TdP degenerating to cardiac arrest. In these patients, a blood sample was obtained within 24 h from TdP/marked QTc prolongation occurrence, and levels of IL-6, TNFα, and IL-1 were evaluated. In all three cases, IL-6 was markedly elevated, ~10 to 100 times more than reference values. Moreover, one patient also showed high circulating levels of TNFα and IL-1. In conclusion, active CIA may represent a currently overlooked QT-prolonging risk factor, potentially contributing in the presence of other “classical” risk factors to TdP occurrence. In particular, a relevant role may be played by elevated circulating IL-6 levels *via* direct electrophysiological effects on the heart. This fact should be carefully kept in mind, particularly when recognizable risk factors are already present and/or the addition of QT-prolonging drugs is required.

## Introduction

Torsades de pointes (TdP) is a polymorphic ventricular tachycardia exclusively occurring in patients with long QT syndrome (LQTS). TdP usually develops in patients presenting a marked QTc prolongation (>500 ms) and is life-threatening, as it can degenerate into ventricular fibrillation (VF) and sudden cardiac death (SCD). In most TdP cases, multiple QTc-prolonging factors need to be simultaneously present, most often QT-prolonging drugs and electrolyte imbalances (1). Other currently known causes of acquired LQTS and TdP include structural heart diseases, bradyarrhythmias, endocrine disorders, autoimmunity (anti-Ro/SSA antibodies), liver diseases, nervous system injuries, HIV infection, starvation, hypothermia, and toxins (1–3).

Mounting recent evidence indicates that in chronic inflammatory arthritis (CIA), particularly rheumatoid arthritis (RA), QTc prolongation is relatively frequent and correlates with the degree of systemic inflammatory activation (4–10). Intriguingly, many basic studies point to direct electrophysiological effects of inflammatory cytokines on the cardiomyocyte action potential duration (APD) (4, 11–19). Moreover, it has been demonstrated that in RA patients, the risk of SCD is significantly increased when compared to non-RA subjects (4, 20). Conversely, to date no data are available about the prevalence of TdP in CIA, and only few cases reporting TdP occurrence in RA patients were described (21–28). Notably, in all cases, RA was considered only an incidental concomitant disease, not a contributing factor to TdP development.

We report three CIA patients with active disease and elevated circulating IL-6 levels, developing marked QTc prolongation, in two cases complicated with TdP degenerating to cardiac arrest.

## Background

Local Ethical Committee approved the study, and patients gave their oral and written informed consent in accordance with the Principles of the Declaration of Helsinki.

### Patient 1

**Figure 1:**
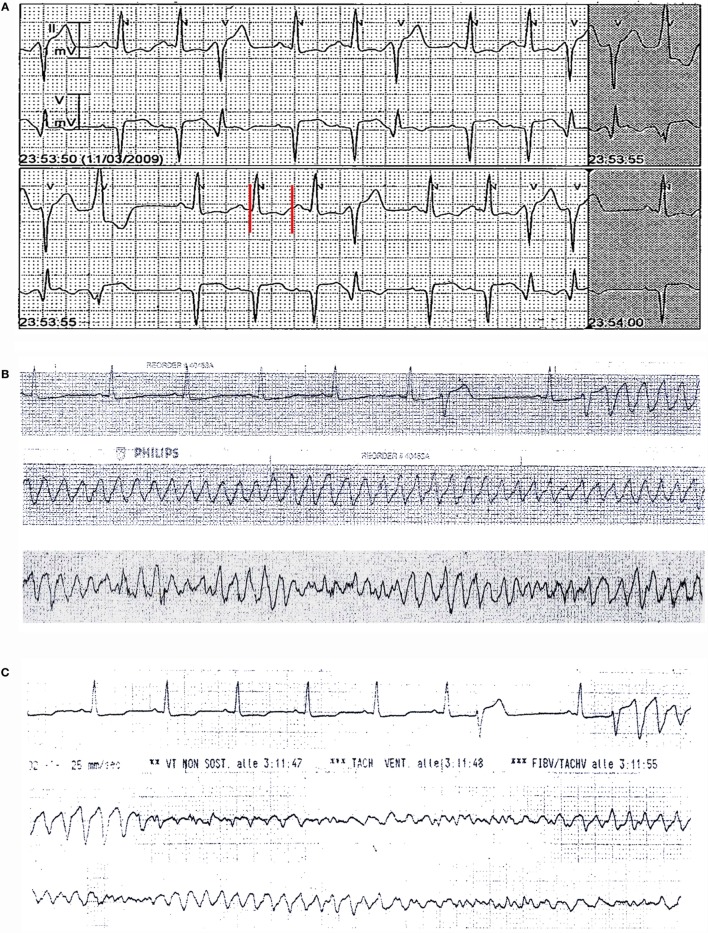
**ECG monitoring findings in patient 1**. **(A)** On admission: frequent polymorphic ventricular ectopic beats with couplets and triplets and QTc prolongation (520 ms). **(B,C)** Recurrent episodes of torsades de pointes, with degeneration to ventricular fibrillation **(C)**. Red vertical lines show QT interval.

A 53-year-old man with no previous cardiac history was admitted for exertional chest pain and dyspnea for few days. His medical history was significant for a poorly controlled type II diabetes mellitus and long-standing RA. Few weeks before, the patients was hospitalized in a Rheumatologic Unit for disease reactivation, and immunosuppressive therapy was started (high-dose subcutaneous methotrexate plus leflunomide) (Tables 1 and 2). Current medications also included metformin and glibenclamide. The body mass index (BMI) was 31.1 (class I obesity). The ECG on admission showed T-wave inversion in peripheral and precordial left leads and QTc prolongation (520 ms), as well as frequent polymorphic ventricular ectopic beats (Figure 1A). Laboratory investigation revealed elevation in cardiac enzymes (troponin I 5.07 ng/mL, r.v. <0.07) and brain natriuretic peptide (1,091 ng/L; r.v. <100), increased A1c (glycated hemoglobin) (9.3%, r.v. 4.3–5.9), and moderate hypocalcemia (7.9 mg/dL, r.v. 8.8–10.5). Potassium and magnesium were normal. Inflammatory markers were elevated [C-reactive protein (CRP) 5.55 mg/dL, r.v. <0.5; erythrocyte sedimentation rate (ESR) 67 mm/h, r.v. <25; fibrinogen 508 mg/dL, r.n. 170–410], and both rheumatoid factor and anti-Ro/SSA antibodies tests were positive (Tables 1 and 2). A complete blood count showed neutrophilia (white blood cell count: 9,500 × 10^3^/μL, neutrophils 78%), with normal values of hemoglobin (12.0 g/dL; mean corpuscular volume 91 fL) and platelets (277 × 10^3^/μL). Echocardiography documented severe left ventricular dysfunction [ejection fraction (EF) ~20%], and coronary angiography demonstrated critical three vessel disease. The patient was treated with diuretics and vasodilators, and then underwent triple coronary artery bypass grafting surgery. Although the immediate postoperative period was uneventful, 3 days later, he suddenly presented with two episodes of TdP, the second one rapidly degenerating to VF (Figures 1B,C) that was reversed by DC-shock. Intravenous magnesium sulfate and calcium gluconate were started, and coronary angiography was repeated. Since normal function of coronary grafts was demonstrated, a residual critical stenosis of the right coronary artery was treated by angioplasty and stenting. Nevertheless, a few hours later, further episodes of TdP with VF degeneration occurred, and the patient underwent temporary ventricular pacing. In the next days, although ventricular arrhythmias did not recur, a permanent implantable cardioverter defibrillator was implanted. Notably, the recently introduced immunosuppressive treatment was maintained during hospitalization, and the patient showed a progressive normalization of the inflammatory activation, as demonstrated by the serial assessment of fibrinogen levels (500, 448, and then 415 mg/dL before discharge). Long-term follow-up demonstrated the improvement of left ventricular function with recurrence of neither life-threatening arrhythmias nor ischemic events. Moreover, RA treatment maintained the disease in remission, and QTc stably remained near-normal/slightly prolonged (450–460 ms) (Figure S1 in Supplementary Material).

**Table 1:** **Demographic, electrocardiographic, and clinical characteristics of patients by case**.

Patient	Age	Gender	QTc (ms)	TdP	Cardiac arrest	Concomitant QTc-prolonging factors	
						Non-pharmacologic	Drugs
1	53	♂	520	Yes	Yes	Acute coronary syndrome, heart failure, diabetes mellitus (type II), hypocalcemia, anti-Ro/SSA	–
2	87	♀	550	Yes	Yes	Heart failure, complete AVB, hypothyroidism, chronic kidney disease	–
3	82	♀	870	ND	No	Acute coronary syndrome, left ventricular hypertrophy, diabetes mellitus (type II), chronic kidney disease, hypokalemia, hypocalcemia, hypomagnesemia	Escitalopram, quetiapine, rivastigmine, trazodone

*TdP, torsades de pointes; AVB, atrioventricular block; anti-Ro/SSA, anti-Ro/SSA antibodies; ND, not documented*.

**Table 2:** **Disease-associated and inflammatory parameters of patients by case**.

Patient	Disease	Ongoing treatment (dosage)	Rheumatoid factor	ESR (mm/h)	Fibrinogen (mg/dL)^a^	CRP (mg/dL)	Plasma cytokine levels (pg/mL)		
							IL-6	TNFα	IL-1
1	Rheumatoid arthritis	Methotrexate (20 mg/week) + leflunomide (20 mg/day)	Positive	**62**	**508**	**5.55**	**26.24**	**81.35**	**5.95**
2	Rheumatoid arthritis	Prednisone (6.25 mg/day)	Positive	**82**	**722**	**1.21**	**9.19**	0.37	0.11
3	Psoriatic arthritis	Methylprednisolone (4 mg/day)	Negative	NA	**600**	**5.53**	**49.54**	1.64	0.24

*ESR, erythrocyte sedimentation rate (reference values: <25 mm/h); CRP, C-reactive protein (reference values: <0.5 mg/dL); IL-6, interleukin-6 (reference values: 0.49–1.25 pg/mL)^b^; TNFα, tumor necrosis factor alpha (reference values: 0.6–3.24 pg/mL)^b^; IL-1, interleukin-1 (reference values: 0.08–0.29 pg/mL)^b^; NA, not available*.

*Bold indicate parameters above reference values*.

*^a^Fibrinogen reference values: 170–410 mg/dL*.

*^b^Cytokine level range measured in an internal reference group of healthy controls*.

### Patient 2

**Figure 2:**
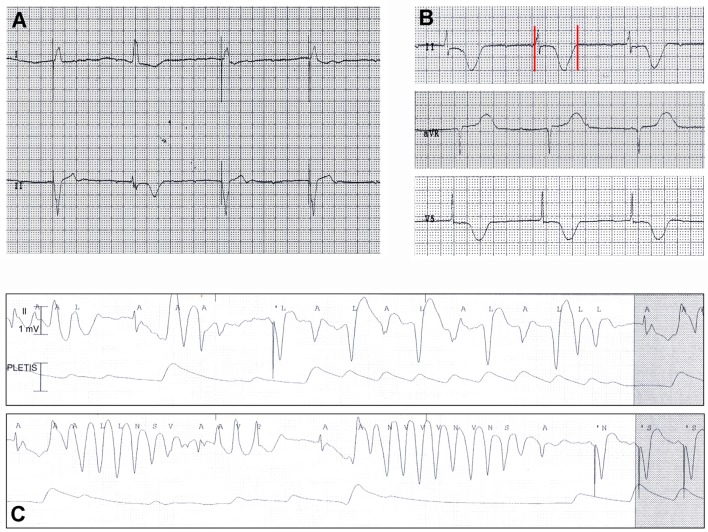
**ECG findings in patient 2**. **(A,B)** Atrioventricular dissociation with low-rate PM-induced ventricular beats (45 bpm), alternating with narrow beats from a junctional escape revealing QTc prolongation (550 ms). Red vertical lines in lead II show QT interval. **(C)** Torsades de pointes episode at ECG monitoring.

An 87-year-old woman developed an out-of-hospital cardiac arrest during the nighttime and was successfully treated by defibrillation. The medical history included hypertension, heart failure, complete atrioventricular block, paroxysmal atrial fibrillation, previous single-chamber pacemaker (PM) VVI implantation (Vitatron C10 S), cerebrovascular disease, chronic kidney disease, and hypothyroidism. Moreover, she had a history of RA that was poorly controlled by steroid treatment. Current medications were warfarin, furosemide, amlodipine, atenolol, l-thyroxine, and prednisone. The ECG on admission demonstrated atrioventricular dissociation with low-rate PM-induced ventricular beats (45 bpm), alternating with narrow beats from a junctional escape revealing significant QTc prolongation (550 ms, Figures 2A,B). Bradycardia was due to activation of “sleep-function,” an algorithm aimed to reduce ventricular pacing by lowering nocturnal pacing rate. Laboratory values were remarkable for inflammatory activation (CRP 1.21 mg/dL, ESR, 82 mm/h, fibrinogen 722 mg/dL) (Table 2) and positive rheumatoid factor, while anti-Ro/SSA antibodies were negative and electrolytes were normal. A complete blood count showed increased white blood cell count (15,900 × 10^3^/μL) with neutrophilia (neutrophils 88%), low hemoglobin values (11.6 g/dL; mean corpuscular volume 91 fL), and normal platelet count (268 × 10^3^/μL). Echocardiography revealed left ventricular hypertrophy (LVH) and moderate systolic dysfunction (EF~40%) with PM-induced ventricular septum dyssynchrony. Shortly after admission, ECG monitoring showed repeated episodes of TdP (Figure 2C) treated with DC-shock. Intravenous magnesium sulfate was started, and PM was reprogramed with a stimulation rate of 110 bpm. As a result, electric storm ceased, and the patient’s clinical condition rapidly improved. In the next days, no further arrhythmic events were recorded. Upgrading to dual-chamber PM was performed, and the patient was discharged with the recommendation to follow-up with a rheumatologist to optimize the immunosuppressive therapy for the underlying RA.

### Patient 3

**Figure 3:**
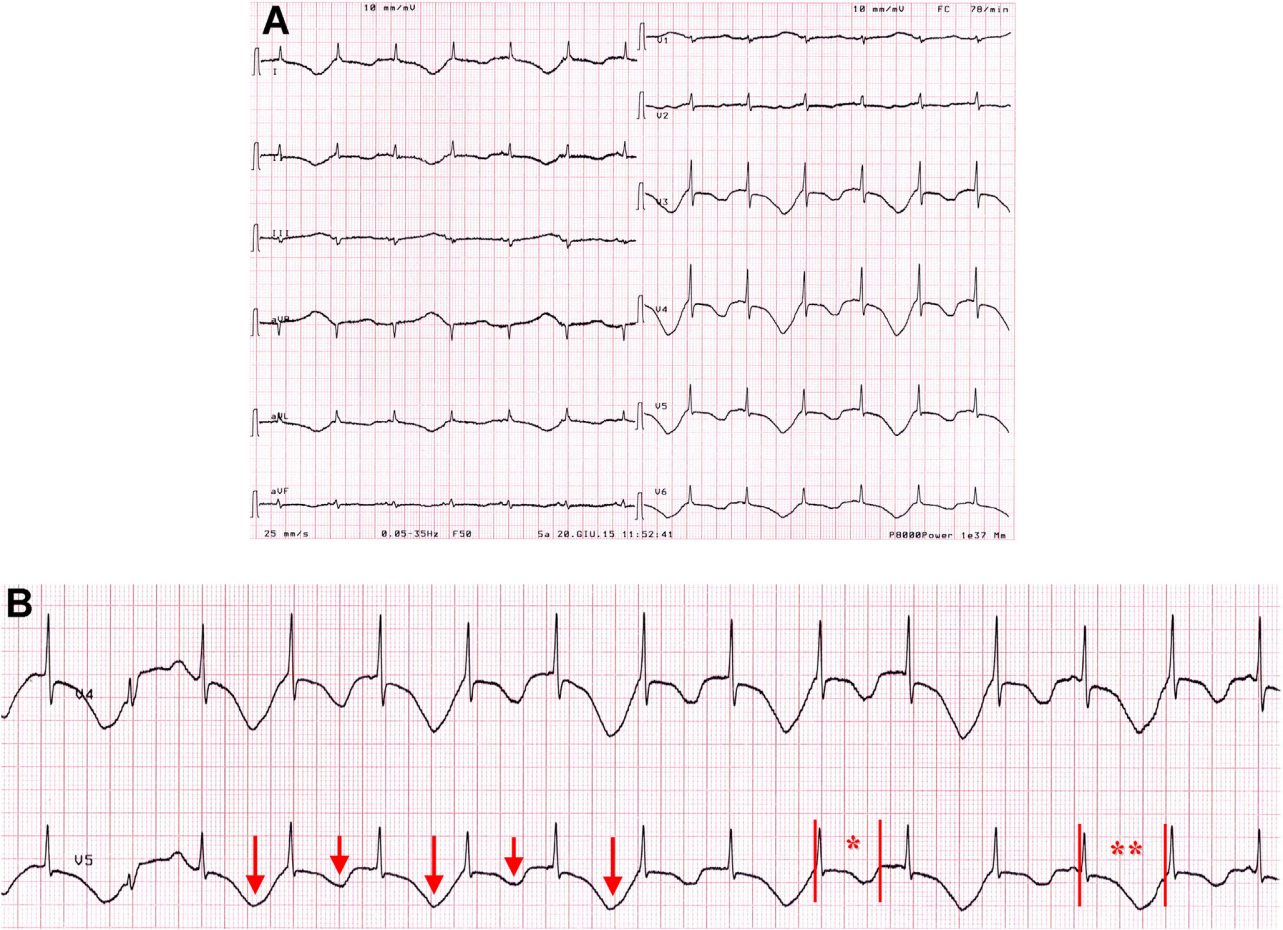
**ECG findings on patient 3 on admission**. **(A,B)** T-wave alternans with marked QTc prolongation, ranging from 690 to 870 ms. Red arrows, long and short, indicate T-wave changes. Red vertical lines in lead V5 show QT interval, while red asterisks indicate where QTc is calculated (*690 ms; **870 ms).

An 82-year-old woman was admitted for palpitations and dizziness. Moreover, it was reported that out-of-hospital ECG monitoring showed runs of ventricular tachycardia (no documentation available). Her medical history included hypertension, Alzheimer’s disease, diabetes mellitus (type II), chronic kidney disease, glaucoma, and breast cancer. She also had a history of psoriatic arthritis (PsA) for several years that was effectively controlled with corticosteroids, methotrexate, and TNF-inhibitors. However, for several months, she was undertreated for renal, ocular, and oncologic conditions. Current medications included ramipril, hydrochlorothiazide, metformin, letrozole, lansoprazole, methylprednisolone, and, among potentially QT-prolonging drugs, quetiapine, escitalopram, rivastigmnine, and trazodone (Table 1). The BMI was 29.6 (overweight). On admission, ECG demonstrated sinus rhythm with sporadic ventricular ectopic beats, diffuse T-wave inversion with T-wave alternans, and marked QTc prolongation (690–870 ms; Figure 3). Blood tests were significant for elevated troponin T levels (227 ng/mL; r.v. <15) and electrolytes imbalance (low levels of potassium, calcium, and magnesium), and high levels of inflammatory markers (CRP 5.53 mg/dL, fibrinogen 600 mg/dL) (Table 2). A1c (glycated hemoglobin) was 7.0%. A complete blood count showed neutrophilia (white blood cell count: 4,100 × 10^3^/μL, neutrophils 76%), low hemoglobin values (9.1 g/dL; mean corpuscular volume 92 fL), with increased platelet count (428 × 10^3^/μL). Echocardiography revealed LVH, regional contraction abnormalities (mid and apical septum) with moderate EF reduction (~40%). No TdP events at ECG monitoring were observed. The patient was treated with aspirin, low molecular weight heparin, diuretics, magnesium sulfate, potassium chloride, and calcium gluconate, while all concomitant QT-prolonging drugs were stopped. In the next days, the patient’s clinical condition progressively improved, and troponin and electrolyte levels normalized. A parallel shortening of the QTc was documented, although it remained significantly prolonged (>550 ms). Since inflammatory marker elevation persisted, corticosteroids and methotrexate were restarted, and at discharge, CRP levels began to reduce (2.39 mg/dL). Notably, at this time also QTc was shortened to ~500 ms (Figure S2 in Supplementary Material).

### Inflammatory Cytokine Measurement

In all patients, a blood sample was obtained within 24 h from TdP/marked QTc prolongation occurrence, and levels of IL-6, TNFα, and IL-1 were evaluated by multiplex assay for cytokine quantification (Bioplex, Bio-Rad, Hercules, CA, USA). In all cases, IL-6 was markedly elevated, ~10 to 100 times higher than reference values, as obtained from an internal reference group of healthy controls. Moreover, patient 1 also showed high circulating levels of TNFα and IL-1 (Table 2).

## Discussion

The main concept emerging from these cases is that CIA may represent a currently overlooked QT-prolonging risk factor, potentially contributing in the presence of other “classical” risk factors to TdP occurrence. More particularly, our study shows that the link found in CIA patients between inflammatory cytokines and QTc prolongation is clinically relevant as it is associated with an actual risk of developing TdP and SCD.

QTc prolongation is relatively common in CIA patients with active disease. In RA subjects, SCD is ~2-times more likely than in the general population, with QTc prolongation representing an independent predictor for all-cause mortality (4, 5, 7, 20). Furthermore, a large population-based study demonstrated that in PsA patients, the incidence of ventricular arrhythmias, including cardiac arrest, is higher than in the general population (29). Although specific epidemiological data are, to date, completely missing, the above considerations strongly suggest that patients with CIA may be at increased risk of TdP. The three cases reported here seem to support this view. In particular, it should be noted that the two RA subjects belong to a larger cohort of 25 consecutive TdP patients prospectively enrolled from 2008 to 2015 at our institution, independent of ongoing therapies and concomitant diseases (2). This corresponds to an 8% incidence of RA in our cohort of TdP, a value 8- to 16-times higher when compared with the expected incidence of RA in the general population, which is ~0.5–1% (30). Consistently, other recent TdP case series reported an ~5% RA incidence, however only considered as incidental (4, 24, 27). In both our cases, TdP rapidly degenerated to cardiac arrest, thus suggesting the possibility that in RA patients, this arrhythmia may be frequently undetected due to a high propensity of progression to SCD in the short-term.

Increasing evidence indicates that the main pathophysiological mechanism underlying CIA-associated QTc prolongation is systemic inflammatory activation, acting both indirectly, by accelerating the development of structural CVD, and directly, by affecting cardiac electrophysiology (4, 10). In particular, many basic studies (11–19) demonstrated that inflammatory cytokines (IL-6, TNFα, IL-1) induce profound changes in the expression and function of potassium and calcium channels resulting in a prolonging effect on cardiomyocyte APD, which translate to QT interval prolongation on the surface ECG. Furthermore, in RA patients, circulating levels of inflammatory cytokines correlated with QTc duration, thus indicating that also *in vivo* these mechanisms are of crucial importance (31). Accordingly, all our patients had active disease with elevated inflammatory markers and cytokine levels. In particular, in all cases, circulating IL-6 was markedly increased, suggesting a particularly relevant role for this molecule in TdP development in these subjects. Experiments in pig ventricular cells demonstrated that IL-6 prolongs APD, by enhancing L-type calcium current (ICaL) (18) Moreover, in RA anti-cytokine therapy with the anti-IL-6-receptor antibody, tocilizumab was associated with a rapid (within 3 months) and significant QTc shortening, which correlated with the decrease in CRP levels (32). Finally, a recent study on a large cohort of women with RA demonstrated that inflammation, as assessed by IL-6 circulating levels, more strongly correlated with fatal than non-fatal cardiovascular events (33). Notably, systemic inflammatory activation occurring in CIA is in many aspects similar to that observed in other chronic inflammatory conditions. Thus, it is highly probable that the reported findings have a more general significance.

Nevertheless, inflammation alone cannot account for marked QTc prolongation observed in our patients. Rather, it probably represented a contributing factor synergistically operating with the other QT-prolonging factors concomitantly present, principally structural heart disease and electrolyte imbalances, and also advanced age and endocrine disorders (diabetes mellitus/metabolic syndrome). In particular, patient 2 was affected with complete atrioventricular block, a condition that *per se* markedly increases TdP risk by inducing electrical ventricular remodeling (34). Patient 1 presented hypocalcemia, and patient 3 combined hypokalemia, hypocalcemia, and hypomagnesemia. Finally, patient 3 was also taking several QT-prolonging drugs, while patient 1 showed circulating anti-Ro/SSA antibodies. These two factors may further reduce the repolarization reserve by inhibiting hERG–potassium channel *via* pharmacologic or autoimmune mechanisms, respectively (1–3).

It is noteworthy that patients 1 and 3 presented with an acute coronary syndrome (ACS). Studies demonstrated that in RA patients, coronary plaques are more inflamed and susceptible to rupture than in non-RA subjects, and the ischemia-driven effects on arrhythmogenesis are well recognized. Accordingly, when compared to non-RA subjects, RA patients’ ACSs show a higher short-term case fatality, and more frequently present with SCD (4, 10). Thus, it is plausible that in these two patients, systemic inflammation may have simultaneously increased myocardial electrical instability both indirectly, by promoting coronary occlusion, and directly by prolonging APD. The specific contribution of the direct cytokine-mediated effects seems to be relevant. Specifically and despite the recovery from myocardial ischemia (and the control of other concomitant risk factors), in both patients 1 and 3, TdP and marked QTc prolongation persisted until disease activity and systemic inflammation were reduced. Recent data from animal models strongly support this view, by providing evidence that inflammatory activation can markedly enhance the effects of acute ischemia in increasing APD duration and ventricular arrhythmia propensity (35, 36).

## Concluding Remarks

In patients with active CIA, the risk of TdP may be higher than expected. A relevant role seem to be played by systemic inflammatory activation, particularly elevated circulating IL-6 levels, *via* direct electrophysiological effects on the heart. Although large prospective studies specifically assessing the actual prevalence of TdP events as well as their contribution to the increased SCD risk observed in these patients are warranted, this fact should be carefully kept in mind, particularly when recognizable risk factors are already present and/or the addition of QT-prolonging drugs is required.

Intriguingly, such mechanisms may be more generally in effect in all the diseases characterized by chronically active systemic inflammation, thereby significantly emphasizing the relevance of our findings.

## Author Contributions

Conception and design of the work: PL. Substantial contributions to the acquisition of data for the work: PL, IB, SL, AS, ES, MRB, and MA. Substantial contributions to the analysis of data for the work: PL, PC, IB, SL, and AS. Substantial contributions to the interpretation of data for the work: PL, PC, IB, GM, ES, DL, NE-S, MB, and FL-P. Drafting the work: PL and PC. Revising the draft of the work critically for important intellectual content: IB, GM, SL, AS, ES, MRB, MA, DL, NE-S, MB, and FL-P. Final approval of the version to be published: PL, PC, IB, GM, SL, AS, ES, MRB, MA, DL, NE-S, MB, and FL-P. Agreement to be accountable for all aspects of the work in ensuring that questions related to the accuracy or integrity of any part of the work are appropriately investigated and resolved: PL, PC, IB, GM, SL, AS, ES, MRB, MA, DL, NE-S, MB, and FL-P.

## Conflict of Interest Statement

We do not have any financial support or other benefits from commercial sources for the work reported on in the manuscript, or any other financial interests, which could create a potential conflict of interest or the appearance of a conflict of interest with regard to the work.

## Supplementary Material

Figure S1 **ECG of patient 1 in the long-term follow-up**. QTc interval is only slightly prolonged (455 ms). Red vertical lines in lead V5 show QT interval.

Figure S2 **ECG findings of patient 3 during hospitalization**. **(A)** Day 7: QTc 580 ms. **(B)** Day 13: QTc 500 ms. Red vertical lines in lead V5 show QT interval.
