# Supplementary figures and images for: Marked QTc Prolongation and Torsades de pointes in Patients with Chronic Inflammatory Arthritis
Source: Front Cardiovasc Med. 2016 Sep 21;3:31. doi: 10.3389/fcvm.2016.00031 (PMC5029147; doi:10.3389/fcvm.2016.00031)

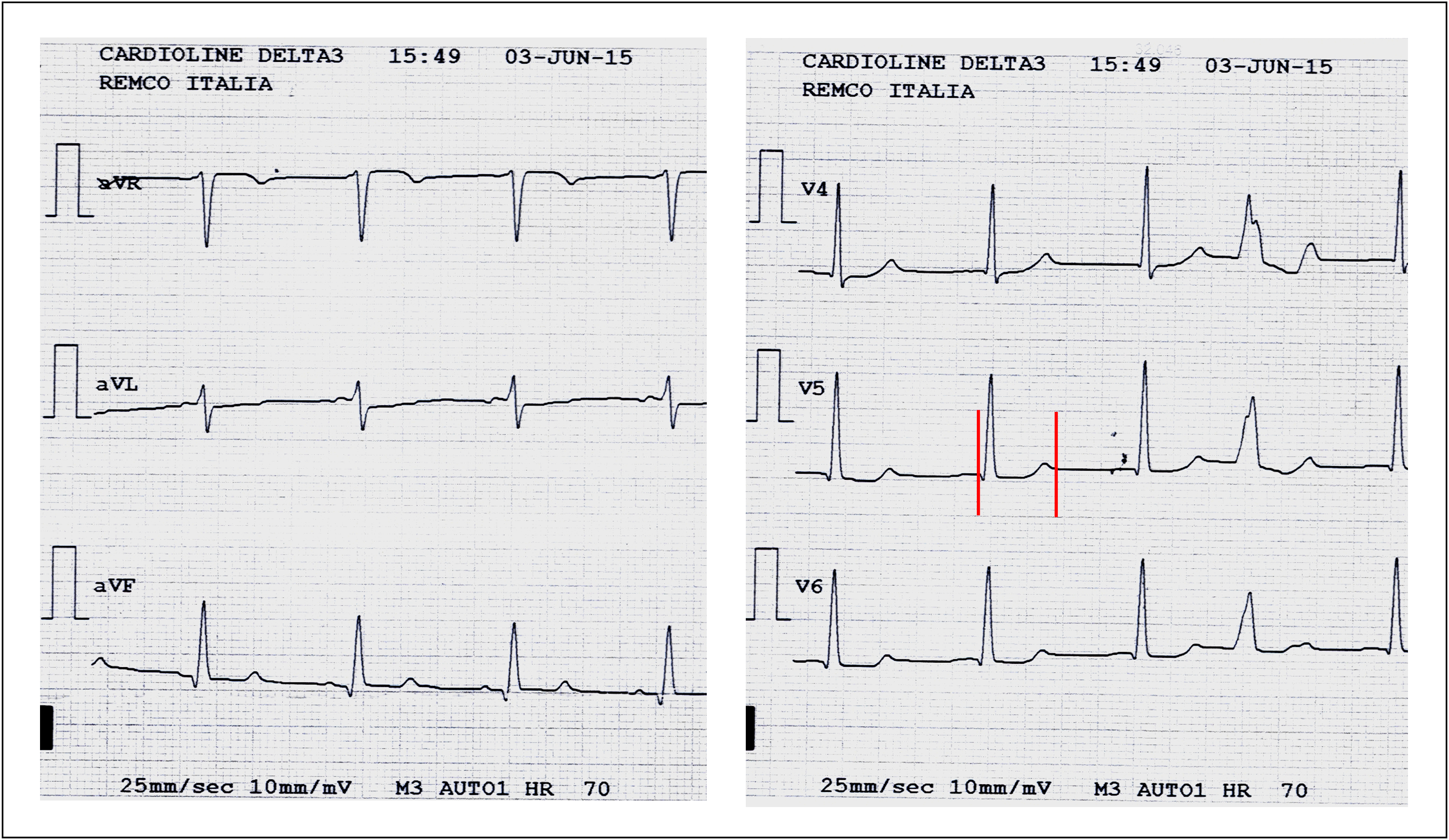

Supplement: Figure S1 — ECG of patient 1 in the long-term follow-up. QTc interval is only slightly prolonged (455 ms). Red vertical lines in lead V5 show QT interval. [file Image_1.TIF]

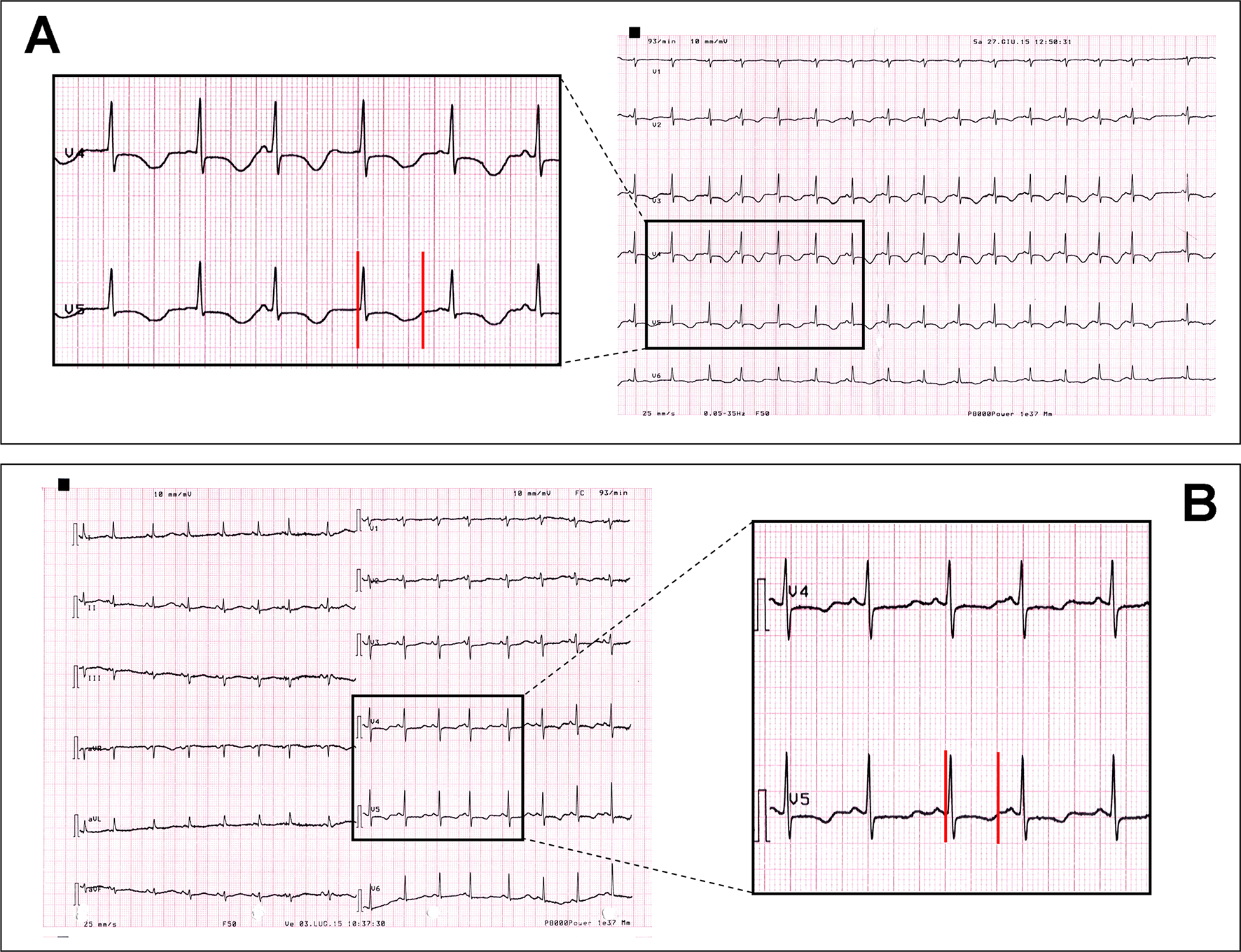

Supplement: Figure S2 — ECG findings of patient 3 during hospitalization. (A) Day 7: QTc 580 ms. (B) Day 13: QTc 500 ms. Red vertical lines in lead V5 show QT interval. [file Image_2.TIF]
